# Distribution and association of antimicrobial resistance and virulence characteristics in *Enterococcus* spp. isolates from captive Asian elephants in China

**DOI:** 10.3389/fmicb.2023.1277221

**Published:** 2023-10-23

**Authors:** Jinpeng Yang, Yanshan Chen, Zhiyou Dong, Wenqing Zhang, Lijuan Liu, Wanyu Meng, Qianlan Li, Keyi Fu, Ziyao Zhou, Haifeng Liu, Zhijun Zhong, Xiao Xiao, Jieyao Zhu, Guangneng Peng

**Affiliations:** ^1^Key Laboratory of Animal Disease and Human Health of Sichuan Province, College of Veterinary Medicine, Sichuan Agricultural University, Chengdu, Sichuan, China; ^2^College of Veterinary Medicine, Yunnan Agricultural University, Kunming, Yunnan, China; ^3^Xishuangbanna Vocational and Technical College, Yunnan, China

**Keywords:** *Enterococcus* spp., Asian elephants, antimicrobial resistance, virulence-associated genes, *Enterococcus faecalis*, *Enterococcus faecium*

## Abstract

*Enterococcus* spp., as an opportunistic pathogen, are widely distributed in the environment and the gastrointestinal tracts of both humans and animals. Captive Asian elephants, popular animals at tourist attractions, have frequent contact with humans. However, there is limited information on whether captive Asian elephants can serve as a reservoir of antimicrobial resistance (AMR). The aim of this study was to characterize AMR, antibiotic resistance genes (ARGs), virulence-associated genes (VAGs), gelatinase activity, hemolysis activity, and biofilm formation of *Enterococcus* spp. isolated from captive Asian elephants, and to analyze the potential correlations among these factors. A total of 62 *Enterococcus* spp. strains were isolated from fecal samples of captive Asian elephants, comprising 17 *Enterococcus hirae* (27.4%), 12 *Enterococcus faecalis* (19.4%), 8 *Enterococcus faecium* (12.9%), 7 *Enterococcus avium* (11.3%), 7 *Enterococcus mundtii* (11.3%), and 11 other *Enterococcus* spp. (17.7%). Isolates exhibited high resistance to rifampin (51.6%) and streptomycin (37.1%). 50% of *Enterococcus* spp. isolates exhibited multidrug resistance (MDR), with all *E. faecium* strains demonstrating MDR. Additionally, nine ARGs were identified, with *tet(M)* (51.6%), *erm(B)* (24.2%), and *cfr* (21.0%) showing relatively higher detection rates. Biofilm formation, gelatinase activity, and α-hemolysin activity were observed in 79.0, 24.2, and 14.5% of the isolates, respectively. A total of 18 VAGs were detected, with *gelE* being the most prevalent (69.4%). Correlation analysis revealed 229 significant positive correlations and 12 significant negative correlations. The strongest intra-group correlations were observed among VAGs. Notably, we found that vancomycin resistance showed a significant positive correlation with ciprofloxacin resistance, *cfr*, and gelatinase activity, respectively. In conclusion, captive Asian elephants could serve as significant reservoirs for the dissemination of AMR to humans.

## 1. Introduction

*Enterococcus* spp., a genus of Gram-positive, spherical or elliptical bacteria, are commonly found in the digestive tracts of humans and animals, and are considered an opportunistic pathogen. Although many strains of *Enterococcus* spp. are harmless commensals, some strains can cause nosocomial and community-acquired human infections, including bacteremia, peritoneal and intra-abdominal infections, and urinary tract infections, among others (Arias and Murray, 2012). The first global mortality estimates for 33 bacterial pathogens and 11 infection types indicate that 539,000 deaths in 2019 were associated with enterococcal infections, with the predominant pathogens being *Enterococcus faecalis* and *Enterococcus faecium*, which account for approximately 80% of enterococcal infections (Ikuta et al., 2022). *Enterococcus* spp. are also used as probiotics, starters in food fermentation, bio-preservatives and indicators of fecal contamination of food or water (Foulquié Moreno et al., 2006; Ben Braïek and Smaoui, 2019). Additionally, *Enterococcus* spp. are important key indicator bacterium in some human and veterinary antimicrobial resistance (AMR) monitoring systems.

*Enterococcus* spp. possess inherent resistance to a range of antimicrobial agents, spanning from β-lactam, cephalosporin, aminoglycoside, to lincosamide, exhibiting varying degrees of susceptibility at different levels (Murray, 1990). They can also acquire resistance to penicillin, chloramphenicol, tetracyclines, and vancomycin through mobile genetic elements carrying antimicrobial resistance genes (ARGs). Additionally, *Enterococcus* spp. play a crucial role in acquiring, storing, and disseminating resistance determinants (Werner et al., 2013; Malik et al., 2022; Sagor et al., 2022). The pathogenesis and biofilm formation of *Enterococcus* spp. are attributed to a multitude of virulence determinants, including aggregation substance (*asa1*), collagen-binding protein (*ace*), enterococcal surface protein (*esp*), gelatinase (*gelE*), hyaluronidase (*hyl*), *E. faecalis* and *E. faecium* endocarditis antigen A (*efaA_fs_* and *efaA_fm_*), pili (*ebpABC* locus, *srt*, and *pil*), the quorum sensing (*fsrA, fsrB* and *fsrC*), and sex pheromones (*cpd*, *cob*, and *ccf*), serine protease (*sprE*), cytolysin (*cylA*, *cylB*, *cylM* and *cylL_L_*; Stępień-Pyśniak et al., 2019b). Biofilms endow *Enterococcus* spp. with enhanced AMR and survival capabilities, while also facilitating their more effective invasion of host and the initiation of infections. Infections related to biofilms of *Enterococcus* spp. are not only difficult to eradicate but also act as centers for bacterial transmission and reservoirs of antibiotic resistance genes (Ch'ng et al., 2019). The process of enterococcal biofilm formation involves the participation of multiple genes, such as *esp., gelE*, *fsrABC*, *ace*, *ebpABC* (Ch'ng et al., 2019). Furthermore, gelatinase and hemolysin enhance its survival and dissemination capabilities within the host, while also inflicting damage on host tissues and the immune system.

The correlation between AMR and virulence traits in *Enterococcus* spp. has been investigated in previous studies (Baylan et al., 2011; Arabestani et al., 2017; Say Coskun, 2019; Alzahrani et al., 2022). In *E. faecium*, there is a certain correlation between ampicillin and vancomycin resistance, where ampicillin-resistant *E. faecium* is often detected prior to vancomycin resistance (Mundy et al., 2000). This suggests that the use of cephalosporins may facilitate the emergence of vancomycin-resistant *E. faecium* (Loeb et al., 1999). Previous studies also have shown that the *vanA* and *erm*(*B*) genes are often located on the same transferable plasmid (Aarestrup et al., 2000). In *E. faecalis*, most of cytolytic strains also express aggregation substance (Chow et al., 1993). In enterococcal clinical isolates, it was found that the *agg* and *fsrB* genes are positively correlated with biofilm formation and gelatinase activity, respectively (Hashem et al., 2017). A positive correlation between gentamicin resistance and hemolysis was demonstrated in *E. faecalis* blood isolates (Huycke et al., 1991). It has been found that a certain correlation between antibiotic resistance and biofilm formation (Fallah et al., 2017). Overall, there may be a potential synergistic interaction between this virulence traits and AMR in pathogenesis or survival. Moreover, there are certain variations in AMR and virulence characteristics among different species of *Enterococcus* spp. *Enterococcus faecalis* exhibits a higher occurrence of virulence factors, including cytolysin, aggregation substance, gelatinase, extracellular superoxide, and extracellular surface protein, compared to *E. faecium*. Conversely, *E. faecium* shows a higher AMR than *E. faecalis* (Jett et al., 1994; Mundy et al., 2000).

In recent years, the monitoring of AMR in wild animals has been performed (Smoglica et al., 2022). Research suggests that wild animals are one of the potential reservoirs for transmitting AMR pathogens to humans, such as wild birds (Stępień-Pyśniak et al., 2019a), non-human primates (Zhu et al., 2021), wild boar (Dias et al., 2022), rodents (Gwenzi et al., 2021). Meanwhile, close contact between humans and animals has been shown to lead to the mutual transmission of antimicrobial resistant microorganisms (van den Bogaard et al., 2001; Kim et al., 2016; Fagre et al., 2022). The recent studies have shown that captive Asian elephants harbor potential pathogenic species as well as a wide range of ARGs (Li et al., 2022; Cao et al., 2023). Being a popular wildlife species, captive Asian elephants have close interactions with humans at tourist attractions, which may lead to the spread of AMR pathogens between humans and animals. To the best of our knowledge, there is currently no research available on AMR and Virulence Characteristics of *Enterococcus* spp. in captive Asian elephants. Therefore, our objective is to determine AMR, ARGs, virulence-associated genes (VAGs), gelatinase activity, hemolysis activity, and biofilm formation, as well as the correlation between them, in *Enterococcus* isolates obtained from captive Asian elephants.

## 2. Materials and methods

### 2.1. Sample collection and processing

From June 2022 to December 2022, a total of 69 fecal samples from elephants were collected from eight sites located in southwestern China, including Kunming Zoo, Yunnan Nationalities Village Ji Xiang Garden, Menglia Shelter, Fengqing Park, Manting Park, Bifengxia Wildlife Park, Jiuding Mountain Wildlife Zoo, and Chengdu Zoo. All captive Asian elephants involved in this study were in a healthy state and did not exhibit any abnormal symptoms. Each elephant was only sampled once for fecal collection. After defecation, fecal samples were collected within 24 h by keepers wearing sterile gloves. The samples were immediately transported with ice bags to the Clinical Veterinary Laboratory at Sichuan Agricultural University, where isolation of *Enterococcus* spp. was performed.

### 2.2. Isolation and identification of *Enterococcus* spp.

*Enterococcus* spp. was obtained from the collected samples utilizing a medium specifically designed for the isolation of *Enterococcus*, as described previously (Stępień-Pyśniak et al., 2019a). The species identification of hypothetical *Enterococcus* spp. isolates was performed by multiplex PCR amplification of the *groES*-*EL* intergenic spacer region and the *E. hirae*-specific muramidase gene (*mur-2*), as described by Holman et al. (2021). Strains were identified as *E. hirae* when both *groES-EL* and *mur-2* exhibited positive amplification. In cases where only *groES-EL* showed positive amplification, the PCR product was subjected to Sanger sequencing at Shanghai Shenggong Biotechnology Co., Ltd. The sequencing results will be analyzed using BLAST on the National Center for Biological Information^1^ to determine the species of *Enterococcus* spp.

### 2.3. Antibiotic susceptibility testing

According to the recommended guidelines by the Clinical and Laboratory Standards Institute (CLSI) in 2020 (M100), antimicrobial susceptibility testing of the isolated *Enterococcus* spp. was performed using the disk diffusion method for 10 antibiotic classes, comprising tetracycline (30 μg; TE), vancomycin (30 μg; VA), ampicillin (10 μg; AMP), rifampin (5 μg; RD), linezolid (30 μg; LZD), erythromycin (15 μg; E), teicoplanin (30 μg; TEC), nitrofurantoin (300 μg; F), chloramphenicol (30 μg; C), streptomycin (300 μg; S), ciprofloxacin (5 μg; CIP), and gentamicin (120 μg; CN). Specifically, the overnight bacterial culture was diluted to a concentration of 0.5 McFarland, and then 100 μL of the diluted suspension was evenly spread on Muller-Hinton agar, followed by incubation at 35°C for 16–18 h. AMR of *Enterococcus* spp. was determined by measuring the size of inhibition zones. *Staphylococcus aureus* ATCC 25923 was used as the quality control strain. *Enterococcus* spp. isolates that exhibit resistance to three or more classes of antibiotics will be defined as multiple drug resistance (MDR). *Enterococcus* spp. isolates that exhibit resistance to at least one class of antimicrobials was defined as antimicrobial resistance (AR).

### 2.4. Identification of ARGs and VAGs

PCR was performed to detect ARGs, including oxazolidinones (*optrA* and *cfr*), tetracyclines [*tet(M)* and *tet(L)*], chloramphenicol (*cat*), erythromycin (*ermA* and *ermB*), ampicillin (*pbp5*), aminoglycosides (*aac(6′)-Ie-aph(2″)-Ia*, *aph(3′)-IIIa*, *ant(6)-Ia*, and *str*), and vancomycin (*vanA* and *vanB*). In accordance with Stępień-Pyśniak et al. (2019b), a total of 23 VAGs in *Enterococcus* spp. were detected by PCR, including *ebpA*, *ebpB*, *ebpC*, *pil*, *srt*, *sprE*, *efaA_fm_*, *efaA*_fs_, *asa1*, *ace*, *esp., gelE*, *hyl*, *cylA*, *cylB*, *cylM*, *cylL_L_*, *fsrA*, *fsrB*, *fsrC*, *cpd*, *cob*, and *ccf*. PCR tests were performed using a 25 μL reaction mixture composed of 12.5 μL of Premix Taq™ (TaKaRa Bio, Otsu, Japan), 8.5 μL of sterile ddH_2_O, 1 μL of each primer, and 2 μL of genomic DNA. The primers, cycling conditions, and amplicon sizes for ARGs are summarized in Supplementary Table 1. Negative controls were used in each PCR run. PCR-positive products were sent to Shanghai Shenggong Biotechnology Co., Ltd. for Sanger sequencing, and the resulting sequences were aligned with reference sequences on NCBI using BLAST.

### 2.5. Phenotypic detection of enterococcal virulence factors

#### 2.5.1. Hemolysin and gelatinase activities

Hemolysin production was assessed by inoculating *Enterococcus* strains onto Columbia agar containing 5% defibrinated horse blood and incubating at 37°C for 24 h. Bacterial colonies displaying transparent hemolytic zones (β-hemolysis) and grass-green hemolytic zones (γ-hemolysis) on agar plates are, respectively, regarded as positive and negative colonies for hemolysin production. *Staphylococcus aureus* ATCC 25923 was utilized as the positive control.

The isolates of *Enterococcus* spp. were inoculated into tubes containing the Nutrient Gelatin (Thermo Scientific™, China). The tubes were incubated at 37°C for 24 to 72 h, followed by a 30-min refrigeration at 4°C. Gelatinase-producing *Enterococcus* spp. still cause liquefaction of the nutrient gelatin medium, even after refrigeration. Conversely, a semisolid consistency of the nutrient gelatin medium after refrigeration indicated a negative result of gelatinase production. *Proteus mirabilis* ATCC®29,906 and *Escherichia coli* ATCC®25,922 were employed as positive and negative controls, respectively.

#### 2.5.2. Biofilm formation assay

The ability of *Enterococcus* spp. to form biofilms was assessed based on a method described by Aladarose et al. (2019). Briefly, after overnight incubation at 37°C on Columbia blood agar plates, a single colony was transferred into tryptic soy broth (TSB) with 0.25% glucose, and incubated overnight at 37°C. The bacterial suspension was adjusted to an optical density (OD_600_) of 0.2–0.257 using TSB. This was then inoculated into a 96-well plate (200 μL per well) with three replicate wells per strain. An additional 200 μL of TSB served as the negative control. After 24 h at 37°C, the wells were washed with PBS, fixed with methanol, and stained with 1% crystal violet dye. Following destaining, the optical density (OD_570_) was measured, and the final OD value for each strain was calculated as the average of three wells. This experiment was conducted in triplicate on separate days. According to previous studies (Stępień-Pyśniak et al., 2019b), the biofilm formation ability of *Enterococcus* spp. can be classified into four levels: negative, weak, moderate, and strong.

### 2.6. Statistical analysis

Statistical analysis was performed using the Fisher’s exact test or Chi-square test in SPSS version 22.0 (IBM Armonk Corp., Armonk, NY, United States), with significance defined as *p* < 0.05. The correlation analysis was performed using the corrplot package in RStudio.

## 3. Results

### 3.1. Isolation and identification of *Enterococcus* spp.

1–2 strains of *Enterococcus* spp. could be isolated from each elephant fecal sample, with no variation in isolation rates across the different regions. A total of 62 strains of *Enterococcus* spp. were isolated from 44 elephant fecal samples, and were then molecularly identified as belonging to 11 different species. The predominant species was *E. hirae* (*17/62, 27.4%*), followed by *E. faecalis* (*12/62, 19.4%*), *E. faecium* (*8/62, 12.9%*), *E. avium* (*7/62, 11.3%*), *E. mundtii* (*7/62, 11.3%*), *E. gallinarum* (*4/62, 6.5%*), *E. casseliflavus* (*3/62, 4.8%*), *E. asini* (*1/62, 1.6%*), *E. flavescens* (*1/62, 1.6%*), *E. malodoratus* (*1/62, 1.6%*), and *E. raffinosus* (*1/62, 1.6%*).

### 3.2. Antibiotic susceptibility test

**Figure 1:**
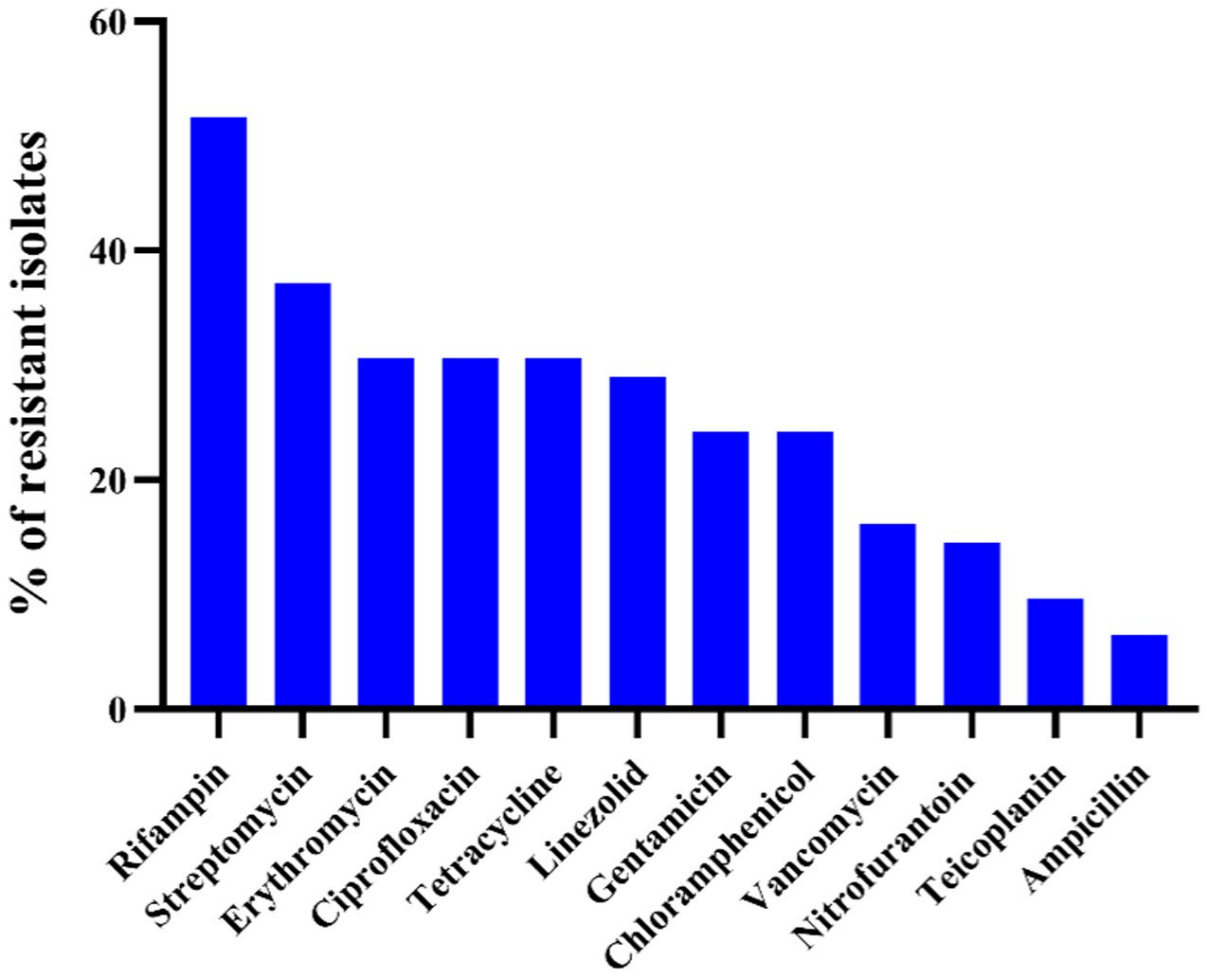
Antimicrobial resistance profile of 62 *Enterococcus* spp. against 12 antibiotics.

The results of the antibiotic susceptibility test of *Enterococcus* spp. isolates are presented in Table 1 and Figure 1. Among the 62 isolates of *Enterococcus* spp., the highest resistance was observed against RD (32/62, 51.6%), followed by S (23/62, 37.1%), TE (19/62, 30.6%), E (19/62, 30.6%), CIP (19/62, 30.6%), LZD (18/62, 29.0%), CN (15/62, 24.2%), C (15/62, 24.2%), VA (10/62, 16.1%), F (9/62, 14.5%), TEC (6/62, 9.7%), and AMP (4/62, 6.5%). *Enterococcus faecalis* was more resistant to RD (91.7%), VA (58.3%), and CIP (50.0%), whereas *E. faecium* was more resistant to CIP (87.5%), RD (75%), and E (62.5%). In 62 strains of *Enterococcus* spp., 53 strains (85.5%) exhibited resistance to at least one antibiotic, 20 strains (32.3%) were resistant to two antibiotics, and the remaining 33 strains (53.2%) were classified as MDR. Notably, the rate of MDR was significantly higher in *E. faecium* (8/8, 100%) when compared other *Enterococcus* species (*n* ≥ 7; *p* < 0.05). There were no significant differences in the prevalence of AR among different species of *Enterococcus* spp. (*p* > 0.5). For *E. hirae*, *E. faecalis*, *E. faecium*, *E. avium,* and *E. mundtii*, the mean multiple antibiotic resistance (MAR) indices were 0.20 (range: 0–0.92), 0.31 (range: 0.08–0.5), 0.39 (range: 0.25–0.58), 0.29 (range: 0–0.83), and 0.15 (range: 0–0.5), respectively.

**Table 1:** Antimicrobial resistance rates of *Enterococcus* spp. from captive Asian elephants.

Antimicrobial agents	No (%) of antimicrobial resistance isolates						
	*Enterococcus hirae* (*n* = 17)	*Enterococcus faecalis* (*n* = 12)	*Enterococcus faecium* (*n* = 8)	*Enterococcus avium* (*n* = 7)	*Enterococcus mundtii* (*n* = 7)	Others (*n* = 11)	Total (*n* = 62)
TE	6 (35.3%)	3 (25.0%)	2 (25.0%)	4 (57.1%)	1 (14.3%)	3 (27.2%)	19 (30.6%)
VA	1 (5.9%)	7 (58.3%)	1 (12.5%)	1 (14.3%)	0	0	10 (16.1%)
AMP	0	0	2 (25.0%)	0	0	2 (18.1%)	4 (6.5%)
RD	6 (35.3%)	11 (91.7%)	6 (75%)	2 (28.6%)	2 (28.6%)	5 (45.5%)	32 (51.6%)
LZD	2 (11.8%)	5 (41.7%)	4 (50%)	2 (28.6%)	2 (28.6%)	3 (27.2%)	18 (29.0%)
E	4 (23.5%)	3 (25.0%)	5 (62.5%)	3 (42.9%)	2 (28.6%)	2 (18.1%)	19 (30.6%)
TEC	2 (11.8%)	3 (25.0%)	0	1 (14.3%)	0	0	6 (9.7%)
F	2 (11.8%)	0	3 (37.5%)	3 (42.9%)	1 (14.3%)	0	9 (14.5%)
C	4 (23.5%)	4 (33.3%)	0	2 (28.6%)	2 (28.6%)	3 (27.2%)	15 (24.2%)
S	8 (47.1%)	2 (16.7%)	3 (37.5%)	2 (28.6%)	2 (28.6%)	6 (54.5%)	23 (37.1%)
CIP	3 (17.6%)	6 (50.0%)	7 (87.5%)	2 (28.6%)	0	1 (9.1%)	19 (30.6%)
CN	3 (17.6%)	1 (8.3%)	4 (50%)	2 (28.6%)	1 (14.3%)	4 (36.4%)	15 (24.2%)
AR	13 (76.5%)	12 (100%)	8 (100%)	6 (85.7%)	5 (71.4%)	9 (81.8%)	53 (85.5%)
MDR	5 (29.4%)	8 (66.7%)	8 (100%)	4 (57.1%)	1 (14.3%)	5 (45.5%)	31 (50%)

TE, Tetracycline; VA, Vancomycin; AMP, Ampicillin; RD, Rifampin; LZD, Linezolid; E, Erythromycin; TEC, Teicoplanin; F, Nitrofurantoin; C, Chloramphenicol; S, Streptomycin; CIP, Ciprofloxacin; CN, Gentamicin; AR, Antimicrobial resistance to at least one category of antimicrobials; MDR, Multiple drug resistance.

### 3.3. Characterization of enterococcal virulence factors

No isolates exhibited β-hemolytic activity, while 9 isolates demonstrated α-hemolytic activity. For gelatinase activities, 15 isolates of *Enterococcus* spp. were found to produce gelatinase, of which 11 strains were identified as *E. faecalis* (73.3%). The gelatinase activity of *E. faecalis* was significantly higher that of other species of *Enterococcus* spp. (*n* ≥ 7; *p* < 0.01).

Overall, among the 62 strains of *Enterococcus* spp., 49 strains (79.0%) were found to be capable of producing biofilms, including 14 strains of *E. hirae*, 12 strains of *E. faecalis*, 5 strains of *E. faecium*, 6 strains of *E. avium*, 4 strains of *E. mundtii*, and 8 strains of other *Enterococcus* species. Among the 49 biofilm-producing *Enterococcus* spp., 36 strains (73.5%) were identified as weak biofilm producers, 10 strains (20.4%) as moderate biofilm producers, and 3 strains (6.1%) as strong biofilm producers. The strong biofilm producers include one strain of *E. mundtii* and two strains of *E. faecalis*. The biofilm-forming ability of *E. faecalis* was significantly higher than other species of *Enterococcus* spp. (*p* < 0.05, Wilcoxon rank-sum test), as all 12 isolates of *E. faecalis* were capable of biofilm formation, with 2 strains identified as strong biofilm producers.

### 3.4. Detection of ARGs and VAGs

**Figure 2:**
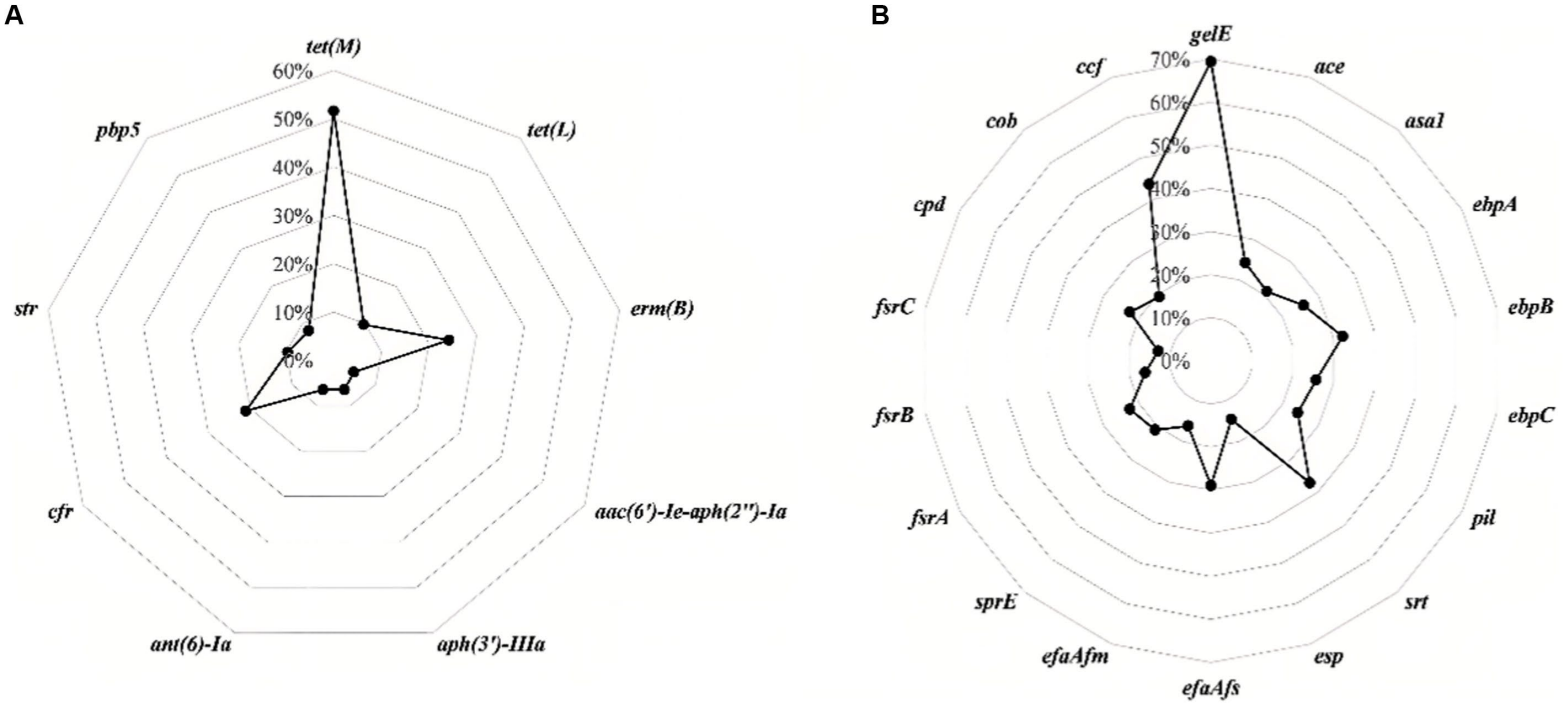
The detection rates of antimicrobial resistance genes **(A)** and virulence-associated genes **(B)** in all isolates of *Enterococcus* spp.

Figure 2 and Table 2 show the detection rates of 23 VAGs and 14 ARGs across all isolates. Overall, 9 out of 14 ARGs were detected in *Enterococcus* spp. isolates. The detection rates of *tet*(*M*) were the highest at 51.6%, followed by *erm*(*B*) (24.2%), *cfr* (21.0%), *str* (9.7%), *tet*(*L*) (9.7%), *pbp5* (8.1%), *aph*(*3*′)-*IIIa* (6.5%), *ant*(*6*)-*Ia* (6.5%), and *aac*(*6′*)-*Ie*-*aph*(*2″*)-*Ia* (4.8%). No *erm*(*A*), *VanA*, *VanB*, *cat*, *optrA* were detected. Interestingly, the detection rates of *cfr* were significantly higher in *E. faecalis* than in *E. faecium* (*p* < 0.01). Conversely, the detection rates of *str* and *pbp5* were significantly higher in *E. faecium* than in *E. faecalis* (*p* < 0.05). The detection rates of *ant(6)-Ia* and *pbp5* were significantly higher in MDR *Enterococcus* spp. than in non-MDR *Enterococcus* spp. (*p* < 0.05).

**Table 2:** Distribution of antimicrobial resistance genes and virulence-associated genes among different *Enterococcus* spp.

Genes	No (%) of antimicrobial resistance genes and virulence-associated genes						
	*Enterococcus hirae* (*n* = 17)	*Enterococcus faecalis* (*n* = 12)	*Enterococcus faecium* (*n* = 8)	*Enterococcus avium* (*n* = 7)	*Enterococcus mundtii* (*n* = 7)	Others (*n* = 11)	Total (*n* = 62)
*tet(M)*	8 (47.1%)	7 (58.3%)	4 (50.0%)	5 (71.4%)	3 (42.9%)	5 (45.5%)	32 (51.6%)
*tet(L)*	3 (17.6%)	2 (16.7%)	1 (12.5%)	0	0	0	6 (9.7%)
*erm(B)*	2 (11.8%)	4 (33.3%)	3 (37.5%)	2 (28.6%)	2 (28.6%)	2 (18.2%)	15 (24.2%)
*aac(6′)-Ie-aph(2″)-Ia*	0	1 (8.3%)	2 (25.0%)	0	0	0	3 (4.8%)
*aph(3′)-IIIa*	0	1 (8.3%)	0	0	1 (14.3%)	2 (18.2%)	4 (6.5%)
*ant(6)-Ia*	0	1 (8.3%)	0	1 (14.3%)	0	2 (18.2%)	4 (6.5%)
*cfr*	0	12 (100%)	0	0	0	1 (9.1%)	13 (21.0%)
*str*	0	0	3 (37.5%)	2 (28.6%)	1 (14.3%)	0	6 (9.7%)
*pbp5*	0	0	5 (62.5%)	0	0	0	5 (8.1%)
*gelE*	11 (64.7%)	11 (91.7%)	7 (87.5%)	3 (42.9%)	5 (71.4%)	6 (54.5%)	43 (69.4%)
*ace*	0	11 (91.7%)	0	1 (14.3%)	1 (14.3%)	2 (18.2%)	15 (24.2%)
*asa1*	4 (23.5%)	2 (16.7%)	2 (25.0%)	0	3 (42.9%)	2 (18.2%)	13 (21.0%)
*ebpA*	0	12 (100.0%)	0	0	2 (28.6%)	2 (18.2%)	16 (25.8%)
*ebpB*	1 (5.9%)	10 (83.3%)	3 (37.5%)	1 (14.3%)	1 (14.3%)	4 (36.4%)	20 (32.3%)
*ebpC*	0	11 (91.7%)	0	2 (28.6%)	2 (28.6%)	1 (9.1%)	16 (25.8%)
*pil*	0	11 (91.7%)	1 (12.5%)	0	1 (14.3%)	2 (18.2%)	15 (24.2%)
*srt*	2 (11.8%)	12 (100.0%)	2 (25.0%)	0	3 (42.9%)	4 (36.4%)	23 (37.1%)
*esp*	0	6 (50.0%)	0	0	1 (14.3%)	2 (18.2%)	9 (14.5%)
*efaA_fs_*	0	11 (91.7%)	1 (12.5%)	3 (42.9%)	1 (14.3%)	2 (18.2%)	18 (29.0%)
*efaA_fm_*	0	1 (8.3%)	7 (87.5%)	1 (14.3%)	0	1 (9.1%)	10 (16.1%)
*sprE*	0	10 (83.3%)	0	0	1 (14.3%)	2 (18.2%)	13 (21.0%)
*fsrA*	1 (5.9%)	10 (83.3%)	0	1 (14.3%)	1 (14.3%)	1 (9.1%)	14 (22.6%)
*fsrB*	0	9 (75.0%)	0	0	0	1 (9.1%)	10 (16.1%)
*fsrC*	0	7 (58.3%)	0	0	0	1 (9.1%)	8 (12.9%)
*cpd*	0	11 (91.7%)	0	1 (14.3%)	1 (14.3%)	1 (9.1%)	14 (22.6%)
*cob*	0	11 (91.7%)	0	0	0	1 (9.1%)	12 (19.4%)
*ccf*	7 (41.2%)	11 (91.7%)	2 (25.0%)	0	1 (14.3%)	6 (54.5%)	27 (43.5%)

For the 23 VAGs, the detection rate of *gelE* was the highest at 69.4%, followed by *ccf* (43.5%), *srt* (37.1%), *ebpB* (32.3%), *efaA_fs_* (29.0%), *ebpA* (25.8%), *ebpC* (25.8%), *ace* (24.2%), *pil* (24.2%), *cpd* (22.6%), *fsrA* (22.6%), *sprE* (21.0%), *asa1* (21.0%), *cob* (19.4%), *efaA_fm_* (16.1%), *fsrB* (16.1%), *esp* (14.5%), and *fsrC* (12.9%). No *hyl, cylA*, *cylB*, *cylM*, and *cyl_L_* genes were detected in any of the tested isolates. The detection rates of VAGs (*ccf*, *ace*, *pil*, *srt*, *cpd*, *fsrA*, *fsrB, sprE*, *fsrC*, *cob*, *ebpA*, *ebpB*, *ebpC*, *efaA_fs_*, and *esp*) in *E. faecalis* were significantly higher than in other species of *Enterococcus* spp. (*n* ≥ 7; *p* < 0.01). There was no significant difference in the detection rates of VAGs between MDR *Enterococcus* spp. and non-MDR *Enterococcus* spp. (*p* > 0.05).

### 3.5. Correlation analysis

**Figure 3:**
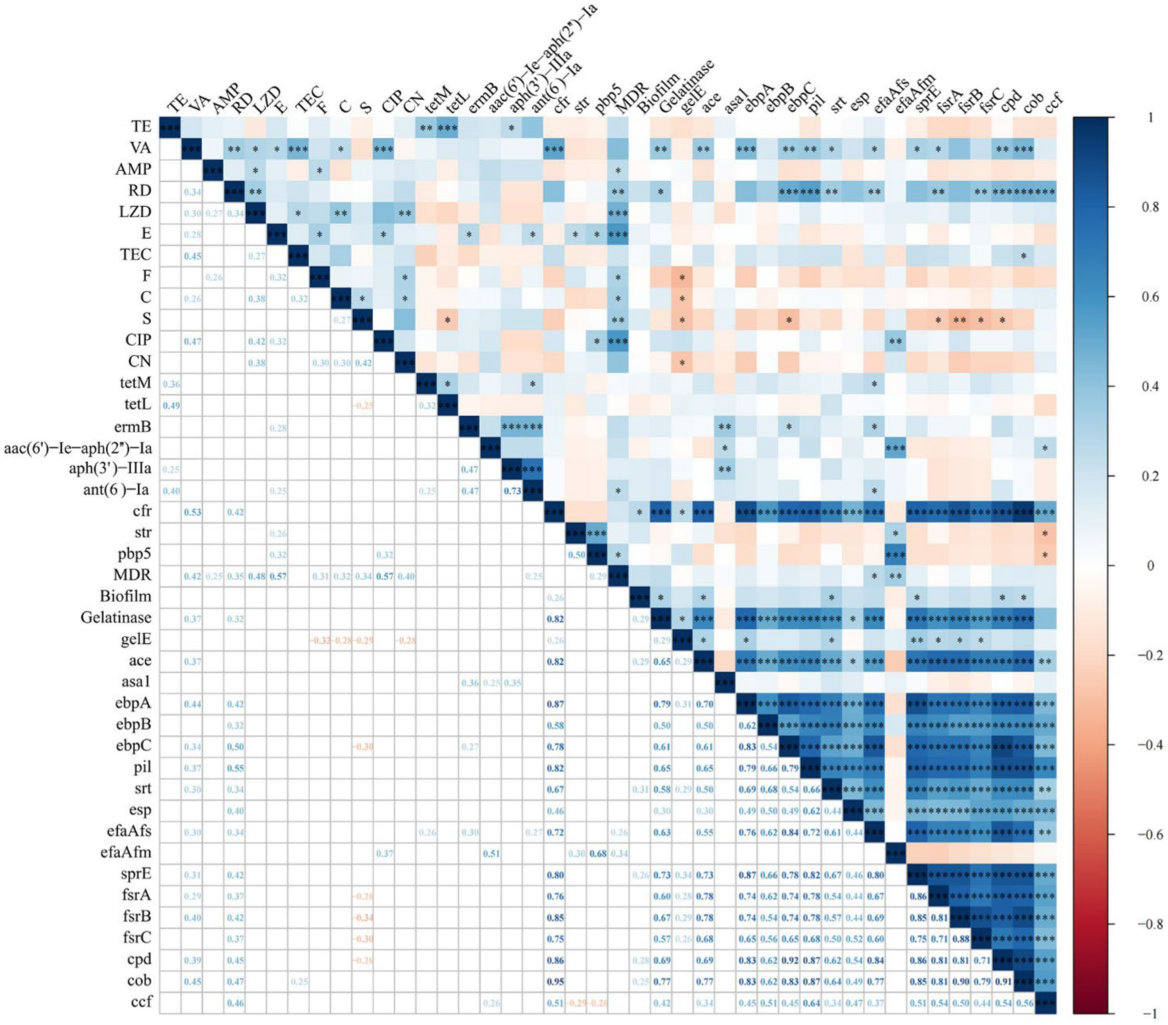
Correlation between antimicrobial resistance, antimicrobial resistance genes, virulence factors, and virulence-associated genes in *Enterococcus* spp. isolates (*, *p* < 0.05; **, *p* < 0.01; ***, *p* < 0.001). The numbers in the heatmap represent the correlation coefficients (*r*) between two objects. Blue color represents positive correlation (*r* > 0), while red color represents negative correlation (*r* < 0).

Figure 3 shows the results of the intragroup and intergroup correlation analysis among AMR, ARGs, VAGs, gelatinase activity, hemolysis activity, and biofilm formation. Correlation analysis within the group of 12 antibiotics revealed 17 pairs of antibiotics showing significant positive correlation and no significant negative correlation. The most significant positive correlation was observed between CIP and VA (*r* = 0.47, *p* < 0.001). A significant correlation was found between gelatinase activity and biofilm formation (*r* = 0.29, *p* < 0.05). There were six pairs of significant positive correlations among ARGs, without any significant negative correlations. The most significant positive correlation was observed between *aph(3′)-IIIa* and *ant(6)-Ia*, both of which determine aminoglycoside resistance (*r* = 0.73, *p* < 0.001). A total of 91 pairs of VAGs exhibited significant positive correlations, while no significant negative correlations were observed. The most significant correlation was found between *ebpC and cpd* (*r* = 0.92, *p* < 0.001). Interestingly, the number of significant correlations within VAGs was significantly higher than that within AMR, ARGs, and virulence factors (*p* < 0.01).

We further analyzed the intergroup correlations. All antibiotics showed a positive correlation with MDR, with significant positive correlations observed between MDR and all antibiotics except for VA, TE, CN and TEC. Only two antibiotics showed a significant correlation with gelatinase activity and biofilm formation, namely VA with gelatinase activity (*r* = 0.37, *p* < 0.01), RD with gelatinase activity (*r* = 0.32, *p* < 0.05). For AMR and ARGs, a total of 9 significantly correlated pairs were identified, with the strongest correlation observed between VA and *cfr* (*r* = 0.53, *p* < 0.001). Further analysis of the correlation between AMR and VAGs revealed 21 significant positive correlations and 9 significant negative correlations. The most significant positive and negative correlations were observed between RD and *pil* (*r* = 0.55, *p* < 0.001), and S and *frsB* (*r* = −0.34, *p* < 0.01), respectively. We only identified two significant correlations between ARGs and MDR (*ant*(*6*)-*Ia* and *pbp5*; *p* < 0.05). ARGs and virulence factors exhibit only two significant correlations: between *cfr* and biofilm formation (*r* = 0.26, *p* < 0.05), and between *cfr* and gelatinase activity (*r* = 0.82, *p* < 0.001). Between ARGs and VAGs, a total of 27 significantly positive correlations and 2 negative correlations were identified. Notably, *cfr* showed significant positive correlations with 16 VAGs, with the strongest correlation observed between *cfr* and *cob* (r = 0.95, p < 0.001). MDR showed significant positive correlations with *efaA_fs_* (*r* = 0.26, *p* < 0.05) and *efaA_fm_* (*r* = 0.34, *p* < 0.01), respectively. For biofilm formation and VAGs, we observed significant positive correlations between five VAGs (*ace*, *srt*, *sprE*, *cpd*, and *cob*) and biofilm formation (all *p* < 0.05). For gelatinase activity and VAGs, we identified significant positive correlations between 15 ARGs and gelatinase activity, with *ebpA* showing the strongest correlation with gelatinase activity (*r* = 0.79, *p* < 0.001).

## 4. Discussion

AMR poses a significant and urgent threat to global public health, ranking among the top 10 global public health threats identified by the World Health Organization. Recent research revealed that bacterial AMR was responsible for 1.27 million direct fatalities in 2019 (Murray et al., 2022). *Enterococcus* spp., as indicators of bacterial AMR, play a crucial role in monitoring the spread of AMR. The interaction between tourists and wild animals may lead to the mutual transmission of AMR. A study demonstrating higher AMR in tourists or local resident relative to that of primates suggests that this high level of AMR may be spreading to primates (Chong et al., 2020). Captive elephants at tourist attractions, being popular animals, frequently come into contact with visitors, which may lead to the transmission of AMR from humans to captive elephants. Therefore, captive Asian elephants may exhibit high levels of AMR. In this study, we characterized AMR and virulence profiles of *Enterococcus* spp. isolated from captive Asian elephants in zoos, while also exploring the correlations between AMR and virulence profiles.

*Enterococcus hirae* was the most common *Enterococcus* species found in the gastrointestinal tract of captive Asian elephants in this study, rather than the commonly observed *E. faecalis* and *E. faecium* in human or other mammalian feces (Lebreton et al., 2014). *Enterococcus hirae* is also the major *Enterococcus* species in the feces of cats (Jackson et al., 2009), and cattle (Jackson et al., 2011), and white-backed stilt (Medeiros et al., 2017). The species differences between elephants and human clinical isolates indicate that elephant-associated *Enterococcus* spp. may not be a significant source or origin of human-associated *Enterococcus* species. In addition to *E. faecalis* and *E. faecium*, other *Enterococcus* species, such as *E. hirae*, *E. avium*, and *E. mundtii*, are deemed infrequent agents of human clinical infections (Ruoff et al., 1990). More *E. faecalis* than *E. faecium* were isolated in this study, possibly due to the higher natural abundance of *E. faecalis* in the gastrointestinal tract (Goh et al., 2017). The majority of clinical enterococcal infections are caused by *E. faecalis* and *E. faecium*, indicating that the transmission of these pathogens from captive Asian elephants to humans could pose a potential risk to human health. However, this study lacks environmental and human samples, making it impossible to determine whether there is a risk of the outward transmission of elephant-associated *Enterococcus* spp.

The intrinsic antibiotic resistance and the capacity to acquire additional antibiotic resistance in *Enterococcus* spp. make infections challenging to manage. This study revealed that *Enterococcus* spp. showed high levels of resistance to RD, which is consistent with the resistance patterns observed in *Enterococcus* spp. previously isolated from pets (Tumpa et al., 2022), ducks (Kissinga et al., 2018), food (Russo et al., 2018), and water environments (Saingam et al., 2021). However, RD in combination with other antibiotics is commonly used to treat *E. faecium* resistant to both VA and LZD (Pankey et al., 2005). This study found VA resistance in 10 strains of *Enterococcus* spp., with 7 of them being specifically identified as *E. faecalis*. However, no VA resistance genes were detected, suggesting that the enhanced VA resistance may be attributed to the biofilm formation of *E. faecalis*. This is consistent with the results of the biofilm formation assay, where 100% *E. faecalis* isolates were identified as biofilm producers. In this study, *E. flavescens*, *E. gallinarum*, and *E. casseliflavus* exhibited intermediate susceptibility to VA, which could be their intrinsic resistance to VA (Vincent et al., 1992). LZD plays a critical role in combating infections caused by vancomycin resistant *Enterococcus* (VRE). Among oxazolidinones, linezolid is notably influenced by the *cfr* gene. Notably, all 12 *E. faecalis* isolates in this study carried the *cfr* gene, with 10 strains exhibiting resistance or intermediate resistance to LZD. It may pose a potential threat to public health due to *cfr* being a transferable resistant gene. We also found that *Enterococcus* spp. isolates capable of forming strong/moderate biofilms were either intermediate or resistant to LZD, which was also found in the report of Aladarose et al. (2019). Different species of *Enterococcus* spp. also exhibit varying susceptibilities to distinct antibiotics. As demonstrated in this study, *E. faecium*, *E. faecalis*, *E. hirae*, and *E. avium* exhibited the highest resistance to CIP, RD, S, and TE, respectively. *Enterococcus faecium* exhibits higher resistance to AMP compared to *E. faecalis*, and AMP-resistant *E. faecalis* strains have been scarcely reported in animals (Miller et al., 2014; Torres et al., 2018). As observed in this study, *E. faecium* (25.0%) isolates were resistant to AMP, whereas no *E. faecalis* isolates showed resistance to AMP. Conversely, *E. faecium* is more prevalent than *E. faecalis* for intrinsic aminoglycosides resistance (Abat et al., 2016), as demonstrated in this study. Interestingly, all the *E. faecium* isolates in this study were found to be MDR, whereas in *E. mundtii*, *E. hirae*, *E. faecalis*, and *E. avium*, MDR was detected at a lower rate. The inherent tenacity, genomic flexibility, and the widespread use of antibiotics have propelled *E. faecium* to become the predominant MDR species among *Enterococcus* spp. (Zhou et al., 2020). A higher prevalence of MDR isolates in *E. faecium* has also been found in other animals (Chotinantakul et al., 2018; Cui et al., 2020). This demonstrates that *E. faecium* is a major source of AMR in the gastrointestinal tract of captive Asian elephants.

Biofilm serves as a significant virulence factor in *Enterococcus* spp., particularly in the pathogenicity of *Enterococcus* spp. Biofilms can enhance bacterial resistance to antibiotics and anti-phagocytosis, posing significant challenges for infection treatment (Lewis, 2001). In the present study, we found that the proportion of biofilm-producing isolates was significantly higher in *E. faecalis* (100%) compared to *E. faecium* (62.5%). Consistent with most studies, *E. faecalis* exhibited a higher propensity to form biofilms than *E. faecium* (Mohamed and Huang, 2007). The formation of biofilm has been correlated with various environmental and genetic factors. Among them, VAGs (*ace*, *gelE*, *esp., fsrABC*, *ebpABC*, *sprE*, and *asa1*) are associated with the formation of biofilms in *Enterococcus* spp. Indeed, VAGs (*ace* and *sprE*) were found to be associated with biofilm formation in this study. Interestingly, we found a highly significant association (*p* < 0.001) between VAGs (*ace*, *sprE*, *fsrA*, *fsrB*, and *cob*) and the formation of strong/moderate biofilms (data not shown). Gelatinase represents a crucial virulence factor in *E. faecalis*, tightly linked to the formation of biofilms. According to our study, all *E. faecalis* strains with gelatinase activity carried *gelE*, which is consistent with the results reported by other authors (Kim et al., 2016; Stępień-Pyśniak et al., 2019b). The encoding of *gelE* and *sprE* occurs within the same operon, and their expression is regulated by the density-sensing system encoded by the *fsr* locus. Thus, we observed that all gelatinase-positive *E. faecalis* almost carries *gelE*, *sprE* and *fsrABC* genes. Previous studies have shown that *gelE* alone is insufficient to predict enterococcal gelatinase activity unless *Enterococcus* spp. carries *fsrAB* or *fsrB* (Hashem et al., 2017). As discovered in this study, 60.5% of *Enterococcus* spp. that were simultaneously positive for *gelE* and negative for *fsrAB* did not exhibit gelatinase activity. In line with the report of Barbosa et al. (2010), the *efaA_fs_* and *efaA_fm_* genes were detected not only in *E. faecalis* and *E. faecium* but also in a few other *Enterococcus* species. The virulence factors of *Enterococcus* spp., such as cytolysin and adhesive substances, can be transferred through the process of gene exchange. The virulence characteristics of *E. faecalis* in this study were significantly stronger than those of other enterococcal species, indicating that the *E. faecalis* isolated from captive Asian elephants should be considered a potential source of some virulence determinants.

Currently, no research has indicated the reasons for the correlation between virulence characteristics and AMR in *Enterococcus* spp. The most synergistic combinations of various virulence traits and AMR may enhance colonization and tissue invasion in the process of enterococcal infection or facilitate adaptation to environmental changes, thereby promoting survival. In general, statistically significant positive correlations were more prevalent in this study, which differs from the findings observed in *Escherichia coli* (Zhang et al., 2021). We observed the strongest positive associations within the following categories: AMR (CIP and VA), ARGs (*aph(3′)-IIIa* and *ant(6)-Ia*), VAGs (*ebpC* and *cpd*), AMR and MDR (E and CIP), AMR and ARGs (VA and *cfr*), AMR and virulence factors (VA and gelatinase), AMR and VAGs (RD and *pil*), MDR and ARGs (MDR and *pbp5*), virulence factors and ARGs (gelatinase and *cfr*), virulence factors and VAGs (gelatinase and *ebpA*), as well as ARGs and VAGs (*cfr* and *cob*). CIP or nitrofurantoin are viable options for managing uncomplicated urinary tract infections caused by VRE. The strong positive correlation between CIP and VA may be the result of CIP use leading to an increase in strains resistant to CIP and VA. The gene *ant(6)* is frequently identified within a gene cluster *ant(6)-sat4-aph(3′)-III* that is part of Tn5405 and other related transposons (Ramirez and Tolmasky, 2010). Furthermore, a prior investigation revealed that the predominant Aminoglycoside modifying enzyme gene profile detected among clinical isolates of *E. faecalis*, *E. faecium*, and *E. avium* was *ant(6)-Ia* + *aph(3′)-IIIa* (Kobayashi et al., 2001). This could be attributed to the strong correlation between *aph(3′)-IIIa* and *ant(6)-Ia*. E, CIP, and *pbp5* exhibit a highly significant correlation with MDR, indicating that they represent the predominant antibiotic-resistant phenotypes and genotype within MDR *Enterococcus* spp. isolates. Some studies have indicated that VA inhibits gelatinase activity (Hashem et al., 2017). Therefore, VA resistance might potentially lead to an elevation in gelatinase activity. This study observed a significant correlation between *cfr* and *cob*, which differs from the correlation between *cfr* and *asa1* observed within LZD-resistant *E. faecium* strains (Krawczyk et al., 2020). The potential statistical association between ARGs and VAGs may indicate a physical connection of genes on the same genetic element (Diarra et al., 2010). Nevertheless, the correlation presented in this study is based solely on statistical findings, and further research is required to elucidate the underlying mechanisms of this correlation, especially concerning the relationship between biofilm formation and AMR.

## Data availability statement

The original contributions presented in the study are included in the article/Supplementary material, further inquiries can be directed to the corresponding author.

## Ethics statement

The animal study was approved by Sichuan Agricultural University Animal Ethical and Welfare Committee. The study was conducted in accordance with the local legislation and institutional requirements.

## Author contributions

JY: Writing – original draft, Formal analysis, Investigation, Methodology. YC: Investigation, Writing – original draft. ZD: Writing – original draft, Software. WZ: Writing – original draft, Data curation. LL: Funding acquisition, Resources, Supervision, Writing – review & editing. WM: Funding acquisition, Resources, Supervision, Writing – review & editing. QL: Funding acquisition, Resources, Supervision, Writing – review & editing. KF: Funding acquisition, Resources, Supervision, Writing – review & editing. ZiZ: Project administration, Validation, Writing – review & editing. HL: Project administration, Validation, Writing – review & editing. ZhZ: Project administration, Validation, Writing – review & editing. XX: Investigation, Writing – review & editing. JZ: Investigation, Writing – review & editing. GP: Writing – review & editing.

## Footnotes

^1^ https://www.ncbi.nlm.nih.gov
