# Supplementary material for: Distribution and association of antimicrobial resistance and virulence characteristics in Enterococcus spp. isolates from captive Asian elephants in China
Source: Front Microbiol. 2023 Oct 23;14:1277221. doi: 10.3389/fmicb.2023.1277221 (PMC10635408; doi:10.3389/fmicb.2023.1277221)
Supplement: Supplementary file 1 [file Table_1.docx]

Supplementary Material

# Supplementary Table 1. PCR primers used in the amplification of resistance genes.

| Gene | Primers（5ˊ-3ˊ） | Size  (bp) | Annealing temperature  (°C) | Reference |
| --- | --- | --- | --- | --- |
| *aac(6')-Ie-aph(2'')-Ia* | F：CAGAGCCTTGGGAAGATGAAG  R：CCTCGTGTAATTCATGTTCTGGC | 348 | 55 | (Chajęcka-Wierzchowska et al., 2016) |
| *str* | F：TATTGCTCTCGAGGGTTC  R：CTTTCTATATCCATTCATCTC | 646 | 45 | (Ruiz-Ripa et al., 2020) |
| *ant(6)-Ia* | F：CGGGAGAATGGGAGACTTTG  R：CTGTGGCTCCACAATCTGAT | 563 | 55 | (Chajęcka-Wierzchowska et al., 2016) |
| *aph(3')-IIIa* | F：GGCTAAAATGAGAATATCACCGG  R：CTTTAAAAAATCATACAGCTCGCG | 523 | 58 | (Chajęcka-Wierzchowska et al., 2016) |
| *cat* | F：GGATATGAAATTTATCCCTC  R：CAATCATCTACCCTATGAAT | 505 | 50 | (Ruiz-Ripa et al., 2020) |
| *erm(A)* | F：TCTAAAAAGCATGTAAAAGAA  R：CTTCGATAGTTTATTAATATTAGT | 645 | 43 | (Ruiz-Ripa et al., 2020) |
| *erm(B)* | F：GAAAAGGTACTCAACCAAATA  R：AGTAACGGTACTTAAATTGTTTA | 639 | 46 | (Ruiz-Ripa et al., 2020) |
| *tetL* | F：ATAAATTGTTTCGGGTCGGTAAT  R：AACCAGCCAACTAATGACAATGAT | 1077 | 55 | (Alzahrani et al., 2022) |
| *tetM* | F：GTGGACAAAGGTACAACGAG  R：CGGTAAAGTTCGTCACACAC | 406 | 57 | (Alzahrani et al., 2022) |
| *vanA* | F：GCGCGGTCCACTTGTAGATA  R：TGAGCAACCCCCAAACAGTA | 314 | 54 | (Alzahrani et al., 2022) |
| *vanB* | F：AGACATTCCGGTCGAGGAAC  R：GCTGTCAATTAGTGCGGGAA | 220 | 54 | (Alzahrani et al., 2022) |
| *cfr* | F：TGAAGTATAAAGCAGGTTGGGAGTCA  R：ACCATATAATTGACCACAAGCAGC | 746 | 54 | (Kehrenberg and Schwarz, 2006) |
| *optrA* | F：AGGTGGTCAGCGAACTAA  R：ATCAACTGTTCCCATTCA | 1395 | 55 | (Alzahrani et al., 2022) |
| *pbp5* | F：AACAAAATGACAAACGGG  R：TATCCTTGGTTATCAGGG | 779 | 54 | (Alzahrani et al., 2022) |

Alzahrani, O.M., Fayez, M., Alswat, A.S., Alkafafy, M., Mahmoud, S.F., Al-Marri, T., et al. (2022). Antimicrobial Resistance, Biofilm Formation, and Virulence Genes in Enterococcus Species from Small Backyard Chicken Flocks. *Antibiotics (Basel).* 11(3). doi: 10.3390/antibiotics11030380

Chajęcka-Wierzchowska, W., Zadernowska, A., and Łaniewska-Trokenheim, Ł. (2016). Diversity of Antibiotic Resistance Genes in Enterococcus Strains Isolated from Ready-to-Eat Meat Products. *J Food Sci.* 81(11)**,** M2799-m2807. doi: 10.1111/1750-3841.13523

Kehrenberg, C., and Schwarz, S. (2006). Distribution of florfenicol resistance genes fexA and cfr among chloramphenicol-resistant Staphylococcus isolates. *Antimicrob Agents Chemother.* 50(4)**,** 1156-1163. doi: 10.1128/aac.50.4.1156-1163.2006

Ruiz-Ripa, L., Feßler, A.T., Hanke, D., Eichhorn, I., Azcona-Gutiérrez, J.M., Pérez-Moreno, M.O., et al. (2020). Mechanisms of Linezolid Resistance Among Enterococci of Clinical Origin in Spain-Detection of optrA- and cfr(D)-Carrying E. faecalis. *Microorganisms.* 8(8). doi: 10.3390/microorganisms8081155
